# *In Vitro* and *In Vivo* Neutralizing Efficacy of Monoclonal Antibodies Against Sars-Cov-2 Variants in Kidney Transplant Recipients

**DOI:** 10.3389/ti.2024.13272

**Published:** 2024-07-16

**Authors:** Ilies Benotmane, Martin Jungbauer-Groznica, Isabelle Staropoli, Delphine Planas, Océane Dehan, Angela Brisebarre, Etienne Simon-Loriere, Samira Fafi-Kremer, Olivier Schwartz, Timothée Bruel, Sophie Caillard

**Affiliations:** ^1^ Department of Nephrology Dialysis and Transplantation, Strasbourg University Hospital, Strasbourg, France; ^2^ Institut national de la santé et de la recherche médicale (Inserm) UMR S1109, LabEx Transplantex, Fédération de médecine translationnelle de Strasbourg (FMTS), Université de Strasbourg, Strasbourg, France; ^3^ Institut Pasteur, Université Paris Cité, Centre national de la recherche scientifique (CNRS) UMR3569, Virus and Immunity Unit, Paris, France; ^4^ Antiviral Activities of Antibodies Group, Université Paris Cité, Centre national de la recherche scientifique (CNRS) UMR3569, Paris, France; ^5^ Université Paris Cité, École Doctorale BioSPC 562, Paris, France; ^6^ Institut Pasteur, Université Paris Cité, G5 Evolutionary Genomics of RNA Viruses, Paris, France; ^7^ National Reference Center for Viruses of Respiratory Infections, Institut Pasteur, Paris, France; ^8^ Department of Virology, Strasbourg University Hospital, Strasbourg, France; ^9^ Vaccine Research Institute, Créteil, France

**Keywords:** sotrovimab, COVID-19, immunocompromised, kidney transplant recipients, monoclonal antibodies, SARS-CoV-2

Dear Editors,

Solid organ transplant recipients continue to face a heightened risk of severe COVID-19, despite a decrease in virus virulence since the emergence of Omicron [1]. Managing preventive and therapeutic strategies in this population poses challenges due to their reduced vaccine response, potential drug-drug interactions with nirmatrelvir-ritonavir, and the ability of variants to escape neutralizing monoclonal antibodies (mAbs) [2, 3]. Neutralization is a surrogate marker of protection for both active (from previous infection or vaccination) and passive immunity (from monoclonal antibodies), and it is utilized for immunobridging of newly available therapeutic antibodies [3, 4]. However, its use in optimizing care for immunocompromised patients is rare, partly due to the absence of a well-defined protective threshold. The emergence of SARS-CoV-2 variants that evade neutralization necessitates ongoing evaluation of therapeutic mAbs and provides an opportunity to explore the relationship between neutralization activity and clinical outcomes. Here, we evaluated the *in vitro* neutralizing activity of sotrovimab and other therapeutic mAbs against XBB.1.5, XBB.1.16.1, and XBB.1.9.1 variants. We also retrospectively investigated the neutralization against these variants of sera from kidney transplant recipients (KTR) who received sotrovimab.

Our initial focus was to assess the *in vitro* neutralizing activity of mAbs that had been utilized since the end of 2021 (namely sotrovimab, cilgavimab-tixagevimab, and imdevimab-casirivimab). As controls, we analyzed the neutralizing activity against the ancestral D614G strain. Neutralization of authentic SARS-CoV-2 isolates were performed with the S-Fuse assay as described in Supplementary Material and previously [5]. Sotrovimab exhibited neutralizing activity against the XBB.1.5, XBB.1.16.1, and XBB.1.9.1 variants, albeit at low levels (with ED50 titers of 0.70 μg/mL, 1.18 μg/mL, and 1.41 μg/mL, respectively, as opposed to 0.04 μg/mL against the D614G variant, Supplementary Figure S1). The cilgavimab-tixagevimab combination and imdevimab-casirivimab displayed no discernible neutralizing activity.

Given this weak but consistent *in vitro* activity of sotrovimab against these variants, our subsequent investigation delved into its *in vivo* neutralization, using the same assay and sera retrieved from 18 KTR followed at Strasbourg University Hospital. These patients had received sotrovimab treatment for confirmed COVID-19, and had accessible post-sotrovimab serum samples during BA.1 and BA.2 breakthrough period spanning from January to March 2022. The administration of sotrovimab was conducted intravenously at a dose of 500 mg. The median age of this cohort was 60.5 years (interquartile range [IQR] 45.2–70.2 years). The median time from transplantation to COVID-19 diagnosis was 2.47 years (IQR 0.34–8.54 years). All but one patient had been vaccinated against SARS-CoV-2, but only two of them demonstrated an effective vaccine response with an anti-spike antibody titer above 264 BAU/mL (Supplementary Table S1). The measurement of *in vivo* neutralizing activity was conducted after a median of 34 days (IQR 18–51.5 days) following sotrovimab administration. All patients’ sera displayed significant serum neutralization against the D614G variant, with a median ED50 titer of 7,641 (IQR 934–11,859). In contrast, although a majority of sera exhibited neutralizing activity above the threshold against XBB.1.5. (n = 17/18), XBB.1.16.1 (n = 16/18), and XBB.1.9.1 (n = 17/18) variants, the titers were low and significantly reduced compared to the neutralization titers against D614G, with median ED50 titers of 31 (IQR 26–121, *p* = 0.04), 24 (IQR 14–85, *p* < 0.0001), and 24 (IQR 11–75, *p* < 0.0001) for XBB.1.5, XBB.1.16.1, and XBB.1.9.1 variants, respectively (Figure 1). The neutralizing titers for XBB.1.5, XBB.1.16.1, and XBB.1.9.1 were reduced by a median of 87-fold, 115-fold, and 154-fold, respectively, compared to D614G.

**FIGURE 1:**
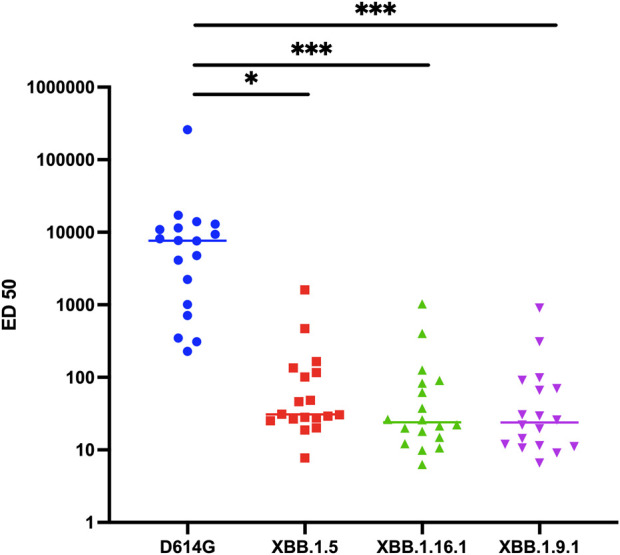
Neutralization activity against D614G, XBB.1.5, XBB.1.16.1, and XBB.1.9.1 variants in sera of COVID-19 kidney transplant recipients (n = 18) receiving Sotrovimab infusion. Results are effective dilution 50% (ED50; titers) as calculated with the S-Fuse assay. Each dot is an individual. Lines indicate medians. The dashed lines indicate the limits of detection. *p = 0.04 according to Friedman test with Dunn’s multiple comparison correction and Spearman non-parametric correlation test. ***p < 0.0001 according to Friedman test with Dunn’s multiple comparison correction and Spearman non-parametric correlation test.

In a subgroup of 10 patients, sera neutralization was assessed before and after sotrovimab administration. After administration of sotrovimab, neutralization activity increased slightly against XBB.1.5 (from a median of 15.61–38.72, *p* = 0.01), XBB.1.16.1 (from a median of 10–26.19, *p* = 0.004), and XBB.1.9.1 (from a median of 10–25.66, *p* = 0.004), Supplementary Figure S2.

Notably, non-hospitalized patients exhibited higher median titers compared to hospitalized patients for each variant: 74.7 (IQR 30.2–142) vs. 26 (IQR 20.4–27.2, *p* < 0.01) for XBB.1.5, 48.4 (IQR 24.2–93.2) vs. 11.3 (IQR 10.2–11.8, *p* < 0.01) for XBB.1.9.1, and 49.8 (IQR 21.6–117.1) vs. 11.4 (IQR 9.0–20.0, *p* < 0.01) for XBB.1.16.1 (Supplementary Figure S3). Advanced age also correlated with lower neutralizing titers against XBB.1.5 and XBB.1.9.1 (Spearman correlation coefficients: −0.49, *p* = 0.04 and −0.54, *p* = 0.02 respectively). None of the other clinical and demographic characteristics were found to be associated with neutralizing titers.

Collectively, the data presented here indicate a persistent *in vitro* and *in vivo* neutralization of sotrovimab against XBB.1.5, XBB.1.16.1, and XBB.1.9.1 variants. These variants are no longer circulating and the current dominant variants (JN.1 and derivatives) fully evades sotrovimab [6]. Nevertheless, our study raises interesting observations on the therapeutic use of mAbs, as it shows a residual antiviral activity of sotrovimab even after its discontinuation from the clinical setting. The minimum dose of mAb necessary to achieve adequate protection has not been established. Data on adintrevimab have shown that a low level of neutralization may be sufficient to provide clinical effectiveness against omicron BA.1 and BA.1.1 [7]. Conversely, during the BA.2 wave, increasing the dosage of tixagevimab-cilgavimab led to a rise in serum neutralizing activity and a decreased risk of COVID-19 breakthrough infections [8]. As higher doses of mAbs administration were found to be safe [9], it may be interesting to consider an increase in the dosage of therapeutic mAbs to boost efficacy against variants harboring partial escape. Indeed, the neutralizing activity against circulating variants is correlated with protection against COVID-19 infection, whether the immunity is passive or active [4]. Furthermore, sotrovimab exhibits an antibody-dependent cellular cytotoxicity (ADCC) activity against the XBB.1.5 variant [5]. Whether such non-neutralizing activities of antibodies contribute to the clinical efficacy of mAbs deserves further investigations. Altogether, our data and the literature suggest that a better mechanistical characterization of antibody activities against variants is needed to optimize patient care.

It is essential to acknowledge the limitations of our study. Being a retrospective, single-center study with a relatively limited sample size and lacking a control group, the findings should be interpreted with caution. We must also consider the potential impact of natural anti-COVID-19 immunity in this population infected with the BA.1 or BA.2 variant. However, it is important to note that the XBB.1.5 variant has the ability to evade antibodies generated after infection by these variant [10].

Providing data on the link between serum neutralization and mAbs efficacy (in our case sotrovimab and XBB.1.5, XBB.1.16.1, and XBB.1.9.1 variants) enables to create a framework to associate neutralization to clinical efficacy over the course of SARS-CoV-2 evolution and to help predict the efficacy of future therapies against future variants.

## Data Availability

The raw data supporting the conclusions of this article will be made available by the authors, without undue reservation.

## Abbreviations

BAU, binding arbitrary units; COVID-19, coronavirus disease 2019; KTRs, kidney transplant recipients; SARS-CoV-2, severe acute respiratory syndrome coronavirus 2; mAbs, monoclonal antibodies.
