# Supplementary material for: In Vitro and In Vivo Neutralizing Efficacy of Monoclonal Antibodies Against Sars-Cov-2 Variants in Kidney Transplant Recipients
Source: Transpl Int. 2024 Jul 16;37:13272. doi: 10.3389/ti.2024.13272 (PMC11286486; doi:10.3389/ti.2024.13272)
Supplement: Supplementary file 1 [file DataSheet1.docx]

**Supplementary Methods**

**1.Neutralization assay**

Neutralization of authentic SARS-CoV-2 isolates were performed with the S-Fuse assay. Briefly, U2OS-ACE2 cells expressing GFP 1-10 and GFP 11 respectively were combined (1:1 ratio) and plated at 2x10^4^ cells per well in a µClear 96-well plates (Greiner Bio-One). Serially diluted mAbs and sera (heat-inactivated for 30 minutes at 56°C) were incubated with indicated SARS-CoV-2 strains for 15 min at room temperature before being added to the U2OS-ACE2 GFP cells. After 18h at 37°C 5% CO2, the cells were fixed with 4% paraformaldehyde, washed with PBS and stained with Hoechst (dilution 1:10,000, Invitrogen). Micrographs were acquired with an Opera Phenix high-content confocal microscope (PerkinElmer) and the number of nuclei and the GFP area were quantified using Harmony software (PerkinElmer). The percentage of neutralization was calculated using the number of syncytia as value with the following formula: 100x(1-(“value with serum”-“value in non-infected”)/(“value in no serum”-“value in non- infected”)). Neutralizing activity against was expressed as the effective dose 50% (ED50; limit of detection: 10).

**2. Viral strains**

The reference D614G and XBB.1.5 strain were previously described.^6,11^ XBB.1.5 was isolated on IGROV-1 cells from a nasopharyngeal swab of an anonymous individual attending the emergency room of Hôpital Européen Georges Pompidou (HEGP; Assistance Publique, Hôpitaux de Paris). XBB.1.9.1 (hCoV-19/France/GES-lPP08594/2023) and XBB.1.16.1 (hCoV-19/France/GES-IPP07712/2023) were supplied by the National Reference Centre for Respiratory Viruses hosted by Institut Pasteur (Paris, France). The human samples, from which the strains hCoV-19/France/GES-IPP08594/2023 and hCoV-19/France/GES-IPP07712/2023 were isolated, were provided from massif des Vosges Hospital and from Laboratory Deux Rives, respectively. Both strains were isolated on Vero-TMPRSS2 and further amplified by one passage on IGROV-1 cells. The viral sequences were deposited on GISAID (D614G: EPI_ISL_414631; XBB.1.5: EPI_ISL_16353849; XBB.1.9.1: EPI ISL 17419152; XBB.1.16.1: EPI_ISL_17383796).

**3. Statistics analysis**

Continuous data are presented as medians and interquartile range and analyzed using the non-parametric Wilcoxon paired test and Spearman non-parametric correlation test. Categorical variables are expressed as counts and percentages and were compared using the Friedman test with Dunn’s multiple comparison corrections. All calculations were performed using GraphPad Prism, version 9.1 (GraphPad Inc., San Diego, CA, USA). A two-sided P value < 0.05 was considered statistically significant.


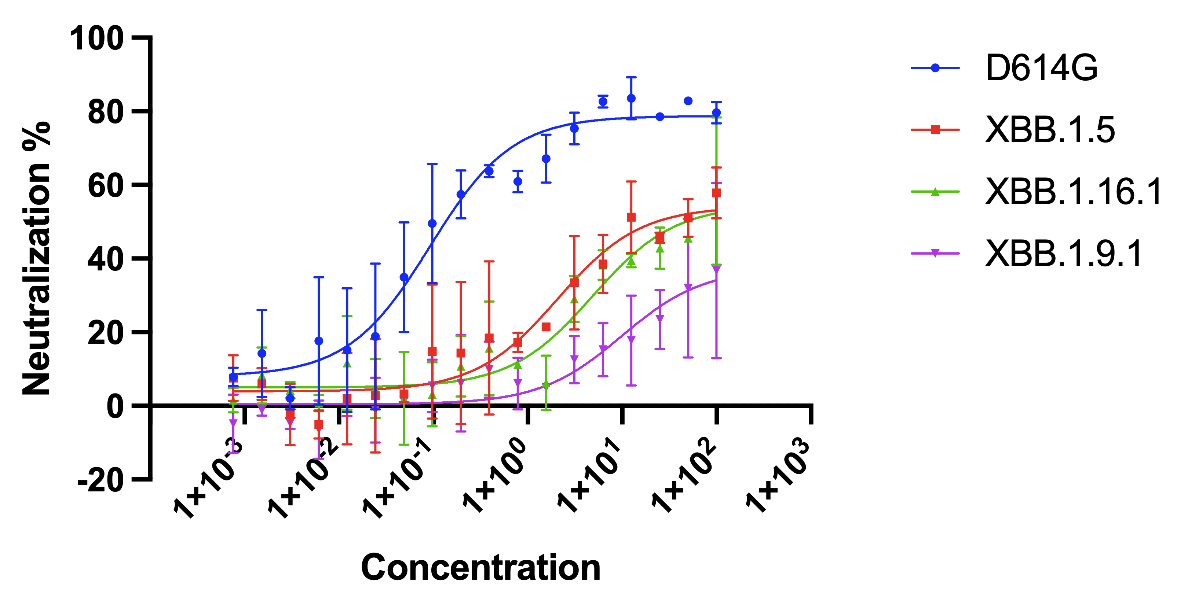


Supplementary Figure 1 : *In vitro* neutralization of sotrovimab against D614G, XBB.1.5., XBB.1.16.1 and XBB.1.9.1. Concentration is expressed in µg/ml. Data are mean ± SD of 3 independent experiments.


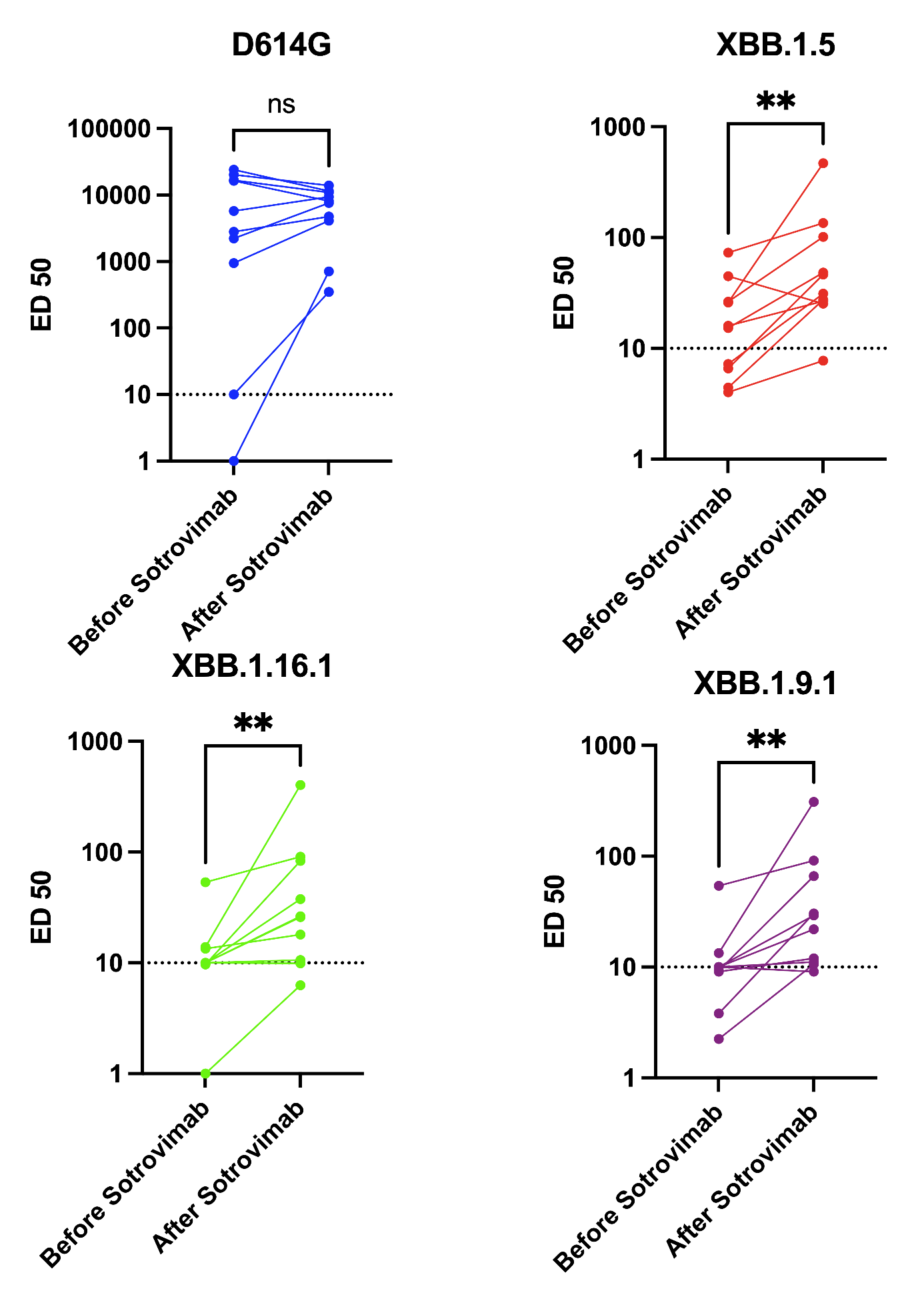


Supplementary Figure 2 : Neutralization of D614G, XBB.1.5., XBB.1.16.1 and XBB.1.9.1 in sera of COVID-19 kidney transplant recipients before and after receiving sotrovimab (n=9). Results are effective dilution 50% (ED50; titers) as calculated with the S-Fuse assay. Each dot represents an individual. The dashed lines indicate the limits of detection. Significance was determined using a two-sided Wilcoxon test (** p<0.01, ns refers to not significant, p=0.56).

**
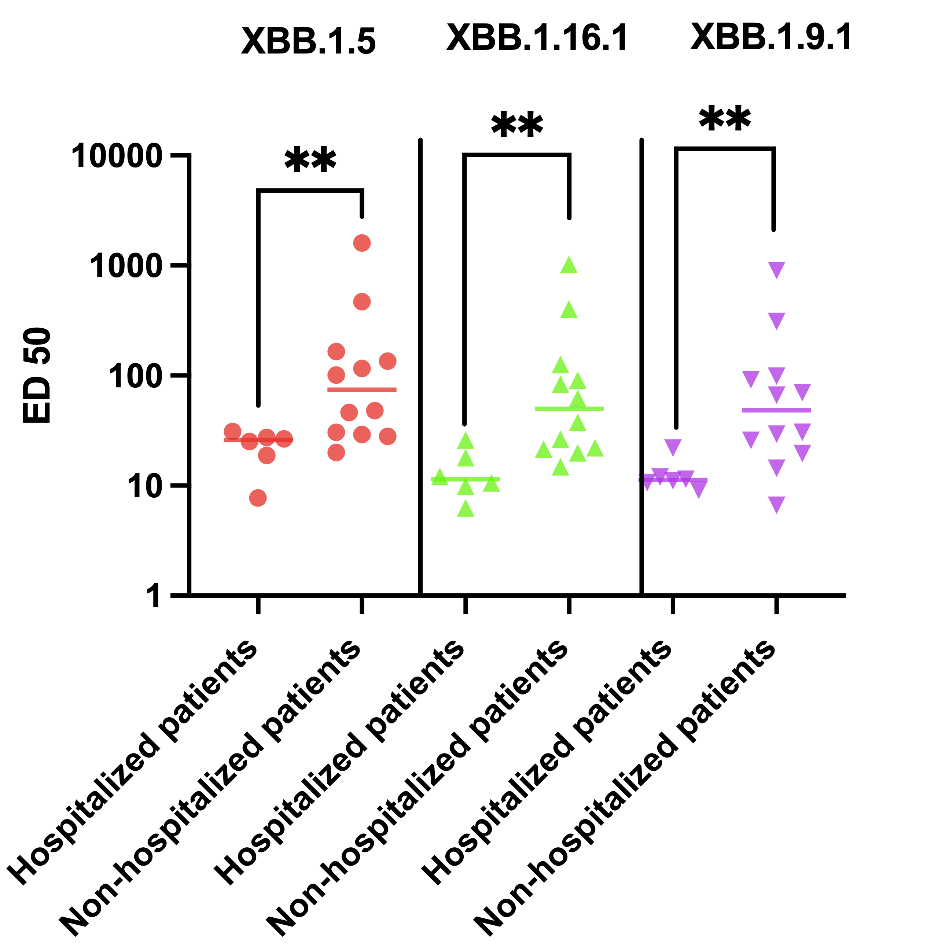
**

Supplementary Figure 3 : Neutralization of XBB.1.5., XBB.1.16.1 and XBB.1.9.1 kinetics in sera of COVID-19 kidney transplant recipients according to hospitalization due to COVID-19 status. Results are effective dilution 50% (ED50; titers) as calculated with the S-Fuse assay. Each dot is an individual. The dashed lines indicate the limits of detection. Significance was determined using a two-sided Mann-Whitney test (** p<0.01).

### Supplementary table 1: Patients’ general characteristics

|  | Total (n=18) |
| --- | --- |
| Age, years | 60.5 [45.2; 70.2] |
| Female | 9 (50%) |
| First kidney transplantation, n, % | 15 (83%) |
| Time from kidney transplantation, years | 3.98 [0.51; 10.5] |
| **Comorbidities** |  |
| Body mass index, kg/m^2^ | 25.2 [23.2; 29.1] |
| Serum creatinine, μmol/L | 121 [92.0; 164] |
| Diabetes | 8 (44%) |
| Cardiovascular disease history | 7 (39%) |
| High blood pressure | 6 (33%) |
| **Immunosuppression** |  |
|  |  |
| Tacrolimus | 14 (78%) |
| Ciclosporine | 2 (11%) |
| Mycophenolate mofetil | 14 (78%) |
| Everolimus | 4 (22%) |
| Belatacept | 1 (5.6%) |
| Corticosteroids | 17 (94%) |
| Azathioprine | 1 (5.6%) |
| **COVID-19 prevention** |  |
| Number of vaccine doses |  |
| 0 | 1 (5.6%) |
| 2 | 2 (11%) |
| 3 | 10 (56%) |
| 4 | 5 (28%) |
| Previous history of COVID-19 | 2 (12%) |
| Pre-exposure prophylaxie with cilgavimab/tixagevimab | 7 (39%) |
| Pre-exposure prophylaxie with imdevimab/casirivimab | 6 (33%) |
| **COVID-19 characteristics and management** |  |
| Asymptomatic COVID-19 | 2 (11%) |
| Mild Covid-19 | 9 (50%) |
| COVID-19–related hospitalization | 6 (33%) |
| Intensive care unit admission | 1 (5.6%) |
| Death | 0 |
| Immunosuppressive lowering | 8 (44%) |
| Dexamethasone | 3 (17%) |
| Time from COVID-19 diagnosis to sotrovimab administration, days | 3.00 [1.00; 4.00] |

Continuous variables are presented as medians (interquartile ranges), whereas categorical variables are presented as counts (percentages).
